# Supplementary material for: Unveiling the Bioactive Potential of the Invasive Jellyfish Phyllorhiza punctata Through Integrative Transcriptomic and Proteomic Analyses
Source: Biomolecules. 2025 Aug 4;15(8):1121. doi: 10.3390/biom15081121 (PMC12383608; doi:10.3390/biom15081121)
Supplement: Supplementary file 1 [file biomolecules-15-01121-s001.zip › Suplementary Material/FileS3/PEPFOLD4/XP_065058654_5390/clusters.html]

 PEP-FOLD clusters


Download archive of all models  here   

|  |  |  |
| --- | --- | --- |
| # | file | sOPEP |
| # cluster 1 - Size: 3 |  |  |
|  | XP\_065058654\_5390-model1 | -14.3627 |
|  | XP\_065058654\_5390-model1.1 | -13.0781 |
|  | XP\_065058654\_5390-model1.2 | -10.4223 |
| # cluster 2 - Size: 9 |  |  |
|  | XP\_065058654\_5390-model2 | -14.1574 |
|  | XP\_065058654\_5390-model2.1 | -12.9797 |
|  | XP\_065058654\_5390-model2.2 | -12.7295 |
|  | XP\_065058654\_5390-model2.3 | -11.47 |
|  | XP\_065058654\_5390-model2.4 | -10.0592 |
|  | XP\_065058654\_5390-model2.5 | -9.52715 |
|  | XP\_065058654\_5390-model2.6 | -9.24753 |
|  | XP\_065058654\_5390-model2.7 | -6.6313 |
|  | XP\_065058654\_5390-model2.8 | -6.36518 |
| # cluster 3 - Size: 19 |  |  |
|  | XP\_065058654\_5390-model3 | -13.4213 |
|  | XP\_065058654\_5390-model3.1 | -13.2757 |
|  | XP\_065058654\_5390-model3.2 | -13.1232 |
|  | XP\_065058654\_5390-model3.3 | -12.7458 |
|  | XP\_065058654\_5390-model3.4 | -12.6196 |
|  | XP\_065058654\_5390-model3.5 | -12.5727 |
|  | XP\_065058654\_5390-model3.6 | -12.2519 |
|  | XP\_065058654\_5390-model3.7 | -12.2458 |
|  | XP\_065058654\_5390-model3.8 | -11.1458 |
|  | XP\_065058654\_5390-model3.9 | -10.0765 |
|  | XP\_065058654\_5390-model3.10 | -10.0075 |
|  | XP\_065058654\_5390-model3.11 | -9.2584 |
|  | XP\_065058654\_5390-model3.12 | -8.50588 |
|  | XP\_065058654\_5390-model3.13 | -7.87749 |
|  | XP\_065058654\_5390-model3.14 | -7.7918 |
|  | XP\_065058654\_5390-model3.15 | -7.47905 |
|  | XP\_065058654\_5390-model3.16 | -7.18325 |
|  | XP\_065058654\_5390-model3.17 | -6.35441 |
|  | XP\_065058654\_5390-model3.18 | -6.03107 |
| # cluster 4 - Size: 7 |  |  |
|  | XP\_065058654\_5390-model4 | -13.289 |
|  | XP\_065058654\_5390-model4.1 | -13.0666 |
|  | XP\_065058654\_5390-model4.2 | -13.0289 |
|  | XP\_065058654\_5390-model4.3 | -12.9459 |
|  | XP\_065058654\_5390-model4.4 | -12.4694 |
|  | XP\_065058654\_5390-model4.5 | -7.91043 |
|  | XP\_065058654\_5390-model4.6 | -7.23128 |
| # cluster 5 - Size: 4 |  |  |
|  | XP\_065058654\_5390-model5 | -13.1794 |
|  | XP\_065058654\_5390-model5.1 | -13.1451 |
|  | XP\_065058654\_5390-model5.2 | -13.1451 |
|  | XP\_065058654\_5390-model5.3 | -12.866 |
| # cluster 6 - Size: 27 |  |  |
|  | XP\_065058654\_5390-model6 | -13.0741 |
|  | XP\_065058654\_5390-model6.1 | -12.9881 |
|  | XP\_065058654\_5390-model6.2 | -12.9881 |
|  | XP\_065058654\_5390-model6.3 | -12.9872 |
|  | XP\_065058654\_5390-model6.4 | -12.9343 |
|  | XP\_065058654\_5390-model6.5 | -12.8105 |
|  | XP\_065058654\_5390-model6.6 | -12.7525 |
|  | XP\_065058654\_5390-model6.7 | -12.7421 |
|  | XP\_065058654\_5390-model6.8 | -12.7141 |
|  | XP\_065058654\_5390-model6.9 | -12.633 |
|  | XP\_065058654\_5390-model6.10 | -12.6242 |
|  | XP\_065058654\_5390-model6.11 | -12.6228 |
|  | XP\_065058654\_5390-model6.12 | -12.5544 |
|  | XP\_065058654\_5390-model6.13 | -12.5302 |
|  | XP\_065058654\_5390-model6.14 | -12.5082 |
|  | XP\_065058654\_5390-model6.15 | -12.5015 |
|  | XP\_065058654\_5390-model6.16 | -12.4958 |
|  | XP\_065058654\_5390-model6.17 | -12.4958 |
|  | XP\_065058654\_5390-model6.18 | -12.4684 |
|  | XP\_065058654\_5390-model6.19 | -12.3861 |
|  | XP\_065058654\_5390-model6.20 | -12.3861 |
|  | XP\_065058654\_5390-model6.21 | -12.3574 |
|  | XP\_065058654\_5390-model6.22 | -12.2809 |
|  | XP\_065058654\_5390-model6.23 | -12.2527 |
|  | XP\_065058654\_5390-model6.24 | -12.2375 |
|  | XP\_065058654\_5390-model6.25 | -12.102 |
|  | XP\_065058654\_5390-model6.26 | -10.6845 |
| # cluster 7 - Size: 6 |  |  |
|  | XP\_065058654\_5390-model7 | -12.8299 |
|  | XP\_065058654\_5390-model7.1 | -12.2253 |
|  | XP\_065058654\_5390-model7.2 | -11.337 |
|  | XP\_065058654\_5390-model7.3 | -10.8291 |
|  | XP\_065058654\_5390-model7.4 | -7.19561 |
|  | XP\_065058654\_5390-model7.5 | -7.15324 |
| # cluster 8 - Size: 9 |  |  |
|  | XP\_065058654\_5390-model8 | -12.6199 |
|  | XP\_065058654\_5390-model8.1 | -11.7975 |
|  | XP\_065058654\_5390-model8.2 | -11.1673 |
|  | XP\_065058654\_5390-model8.3 | -8.10399 |
|  | XP\_065058654\_5390-model8.4 | -8.0939 |
|  | XP\_065058654\_5390-model8.5 | -7.6525 |
|  | XP\_065058654\_5390-model8.6 | -6.24877 |
|  | XP\_065058654\_5390-model8.7 | -6.2138 |
|  | XP\_065058654\_5390-model8.8 | -6.04323 |
| # cluster 9 - Size: 1 |  |  |
|  | XP\_065058654\_5390-model9 | -10.5682 |
| # cluster 10 - Size: 2 |  |  |
|  | XP\_065058654\_5390-model10 | -10.0898 |
|  | XP\_065058654\_5390-model10.1 | -7.58204 |
| # cluster 11 - Size: 1 |  |  |
|  | XP\_065058654\_5390-model11 | -9.72073 |
| # cluster 12 - Size: 1 |  |  |
|  | XP\_065058654\_5390-model12 | -7.36029 |
| # cluster 13 - Size: 1 |  |  |
|  | XP\_065058654\_5390-model13 | -6.67489 |
| # cluster 14 - Size: 4 |  |  |
|  | XP\_065058654\_5390-model14 | -6.59325 |
|  | XP\_065058654\_5390-model14.1 | -6.27528 |
|  | XP\_065058654\_5390-model14.2 | -6.1225 |
|  | XP\_065058654\_5390-model14.3 | -6.0811 |
| # cluster 15 - Size: 2 |  |  |
|  | XP\_065058654\_5390-model15 | -6.53346 |
|  | XP\_065058654\_5390-model15.1 | -6.19086 |
| # cluster 16 - Size: 1 |  |  |
|  | XP\_065058654\_5390-model16 | -6.49125 |
| # cluster 17 - Size: 1 |  |  |
|  | XP\_065058654\_5390-model17 | -6.41139 |
| # cluster 18 - Size: 1 |  |  |
|  | XP\_065058654\_5390-model18 | -6.24351 |
| # cluster 19 - Size: 1 |  |  |
|  | XP\_065058654\_5390-model19 | -6.20062 |
|  |  |  |
